# Consciousness, mindfulness, and introspection: integrating first- and second-person phenomenological inquiry with experimental and EEG data to study the mind

**DOI:** 10.3389/fpsyg.2025.1558453

**Published:** 2025-09-08

**Authors:** Anita Milicevic, Angela Blazely, Anatol Bragin, Ross W. J. Dunseath, Nicholas J. Matiasz, Mauricio Watanabe Ribeiro, Camila Sardeto Deolindo, Elisa H. Kozasa, B. Alan Wallace

**Affiliations:** ^1^Center for Contemplative Research, Crestone, CO, United States; ^2^South Western Sydney Local Health District, Sydney, NSW, Australia; ^3^Macquarie University, Sydney, NSW, Australia; ^4^David Geffen School of Medicine, Brain Research Institute, University of California, Los Angeles, Los Angeles, CA, United States; ^5^Division of Perceptual Studies, School of Medicine, University of Virginia, Charlottesville, VA, United States; ^6^Hospital Israelita Albert Einstein, São Paulo, Brazil; ^7^Santa Barbara Institute for Consciousness Studies, Santa Barbara, CA, United States

**Keywords:** mixed methods research design, first- second- and third-person inquiry, purposive sampling, participant observer, consciousness and introspection study

## Abstract

Studies on consciousness need to develop further a mixed methods research design that effectively integrates first-, second- and third-person research approaches. Mixed methods research has not fully explored the methodological potential of the participant–observer role and the interdependence of knowledge across disciplines to gain a deeper understanding of such inquiries. This paper describes a theoretical and methodological framework that integrates phenomenological, psychological, and electrophysiological methods for the study of consciousness and the mind. This methodological approach includes the purposive sampling of contemplative practitioners extensively trained to observe their mental states and processes with sustained attention, mindfulness, and introspection. By reporting the theoretical and methodological framework here, it is anticipated that the authors' experience of having used this in a small pilot study will offer valuable guidance to fellow researchers seeking to maximize the rigor of their in-depth studies on consciousness and the mind.

## Introduction

Researchers have articulated the importance of Mixed Methods Research (MMR) in understanding the phenomena being studied (Achepohl et al., 2022; Huynh et al., 2019; Mascaro et al., 2022). In this paper, *Mixed Methods Research* (MMR) is used as a comprehensive term to encompass diverse procedures involved in combining, integrating, linking, and applying multiple data collection methods as well as both qualitative and quantitative research approaches (Creswell, 2003; Martiny et al., 2021). This paper illustrates MMR which integrates phenomenological, psychological, and electrophysiological methodologies to explore and investigate consciousness and the mind. A large body of scientific research has highlighted the health benefits of meditation (Gamaiunova et al., 2021; Deolindo et al., 2020; Gotink et al., 2015; Jamil et al., 2023; Josipovic and Baars, 2015; Lawrence et al., 2024; Rich et al., 2022; Ryff, 1989), and whilst MMR has been somewhat widely used in the study of the phenomena (Goyal et al., 2025; Mascaro et al., 2022; Keyworth et al., 2014), there are a number of limitations of the MMR protocols that have been utilized. In spite there being a history of neurophenomenology in mixed method research (Bitbol and Petitmengin, 2017; Berkovich-Ohana, 2017; Huynh et al., 2019; Petitmengin et al., 2019; Varela, 1996, 1999), there remains scant inclusion of comprehensive and reliable first-person and second-person perspectives and how this data has been integrated with third- person research approaches (Northoff and Ventura, 2025; Olivares et al., 2015). There are relatively few instances of employing a phenomenological mixed method where phenomenology informs both qualitative and quantitative data generation, integrated analysis, and interpretation (Martiny et al., 2021). Furthermore, whilst the impact of meditation on health and wellbeing has been widely studied, the application of meditation to the study of the nature of consciousness itself and the mind (rather than its correlated brain functions) has been largely overlooked in the scientific literature. Additionally, if meditation has been used in the study of consciousness, participants are mostly novice meditators (Abdoun et al., 2019; Bordbar et al., 2024; Mascaro et al., 2022) rather than those who have undergone rigorous long-term training in contemplative practices designed specifically to explore the nature of consciousness and the mind itself. Indeed, most scientific studies on consciousness are designed to look at brain function and its relationship with consciousness as well as the epiphenomena of consciousness (Josipovic, 2021, 2024; Laukkonen and Slagter, 2021; Metzinger, 2020, 2024; Sattin et al., 2021).

The theoretical and methodological frameworks and protocols outlined here are intended to introduce an innovative use of Interpretive Phenomenological Analysis and its application to first-and second-person inquiry, and how this can be integrated or synthesized with third-person quantitative data from questionnaires and EEG recordings. Furthermore, these frameworks also explore the potential for the utilization of meditation practices in the scientific study of consciousness and the mind, by including first-and second-person phenomenological data from highly advanced contemplative practitioners. These practitioners have finely trained their attention, introspection and mindfulness to specifically gain insight into the nature of consciousness and the mind itself.

For the purposes of this paper, it is important here to examine what is meant by “consciousness” and “the mind”. The first-person experience of thoughts, emotions, and other mental processes initially provide a direct means for looking at states of consciousness (Wallace, 2012) where “consciousness is not just another object of knowledge, but also, and more fundamental, that by which any object is knowable” (Frank et al., 2024, p. 187). Indeed, consciousness includes a process of awareness as the only mode of observation that can illuminate the nature of mental events themselves (Wallace, 2012). According to Buddhism, the defining characteristics of consciousness are sheer luminosity and cognizance, which are non-conceptual (Wallace, 2011). To our knowledge, the inter-dynamic relationship between consciousness and mental processes has not been explored or investigated in Western psychology and related disciplines.

While contemplative traditions, such as Buddhist psychology, refer to non-referential awareness as an original and unconditioned reality (Kudesia and Nyima, 2015), in Western psychology, the term awareness has often been used to refer to being conscious of something or someone (American Psychological Association, 2025a,b). In Buddhist psychology, this awareness of something, or someone is known as consciousness, which engages with perceptual, cognitive, and affective phenomena. In other words, this distinction between awareness (rig pa) and consciousness (shes pa) is not made in Buddhism. Furthermore, in Buddhism, consciousness and awareness are not considered epiphenomena of neurological processes or brain structures. Based on this view of consciousness and awareness, a contemplative who has been adequately and rigorously trained in attention, mindfulness, introspection, and discernment may let their

…awareness rest in its own nature, witnessing the sheer luminosity and cognizance of awareness itself. When awareness illuminates its own nature, this metacognition is the awareness of awareness (Wallace, 2011, p. 136).

While the mind includes a wide array of mental states and processes, consciousness, as stated previously, is characterized by both luminosity and cognizance. Cognizance refers to *the non-material, space-like quality of consciousness*, which is entirely devoid of color, shape, form, or physical dimensions (Rabten, 2008, p. 31). However, despite its lack of these physical properties, it is not merely an abstract entity like space. Unlike space, luminosity is coupled with the essential characteristic of cognizance. Cognizance is the conscious faculty that enables us to perceive forms and sounds, as well as engage in various forms of reflection, inference, and understanding. While it may be subtle in nature, cognizance is a momentary phenomenon that maintains its own causal continuum (Rabten, 2008).

For the purpose of this theoretical and methodological framework, the mind (Tib. sems, Skt. citta) refers to the dualistic awareness that clings to appearances, conceptually observes its own processes, and arouses pleasure and pain through intellectual fabrications and the acceptance and rejection of virtue and vice (Wallace, 2020).

It is from this stance that the MMR design was developed. In psychological terms, this vigilance—the awareness of awareness—refers to meta-awareness (Frank et al., 2024). The process of meta-awareness, distinguished but coupled with dualistic awareness that conceptually observes its processes in the form of the first- and second person reporting of such experiences, is paramount to MMR designs for studying the nature of consciousness and the mind. Despite the significance of this, such methods still need to be explored in Western psychology, neuroscience, and related disciplines. The aim of the paper is to depict this exploration.

This oversight in MMR design was highlighted more than a century ago by William James (Wallace, 2012). Notably, James (1950/1890) proposed that the study of the mind, including attention, introspection, and cognition, should proceed in a three-fold fashion: (i) behavioral study (through objective, physical, and quantifiable phenomena); (ii) studying the neural correlates that help give rise to subjective experience (neuropsychology); (iii) direct empirical observation of mental phenomena themselves. The first two approaches have been thoroughly researched, while the direct empirical observation of mental phenomena has largely been ignored. It would be imperative to include direct empirical observation of mental phenomena in studies of consciousness using MMR. Accordingly, this would underscore the necessity of reliable and credible introspection in such MMR studies on consciousness. In fact, neuroscientists, psychologists, and physicists collaborated toward the end of the last century to emphasize this necessity (Hut and Shepard, 1996; Frank et al., 2024; Varela, 1996).

The direct empirical observation of mental phenomena has presented somewhat of a conundrum to scientific theory and methods. Whilst the inclusion of first-and second-person perspectives is acknowledged, but not routinely included, exactly how to integrate these perspectives has been problematic. Particularly in light of what is understood by the term introspection, and its application as a scientific tool and subjective skill (Wallace, 2004). Introspection refers to the process of attempting to directly access one's own internal psychological processes or states (American Psychological Association, 2025a,b).

Furthermore, in Buddhist psychology introspection is a mental faculty by which one monitors how one's body, speech, and mind are functioning (Wallace, 2019, 2020), and it is a trainable skill by way of meditation. Thus, it is argued that in this instance Buddhist psychology has quite a bit to offer scientific inquiry, specifically within the context of Western psychology. Indeed, the combination of both Buddhist and Western psychology may prove to be particularly meaningful in the scientific study of the nature of consciousness and the mind. In this context, contemplative practices as a form of meditation in Indo- Tibetan Buddhist tradition can offer unique insights into the subjective experiences and reporting on consciousness and the mind. When this is formally and routinely integrated into MMR, it has the potential to reveal unique insights into the science of consciousness and the mind.

In this context, contemplative practices specifically refer to methods developed to train attention, mindfulness, and introspection. Mindfulness and introspection have a specific definition and involve specific training methods which are rooted in the Indo- Tibetan-Buddhist tradition.

Asanga, an Indian Buddhist scholar and contemplative in the 5th century C.E., emphasized that mindfulness prevents the mind from wandering, while introspection identifies when this wandering occurs. Asanga states, “[Mindfulness] is non-forgetting by the mind (citta) with regard to the object experienced. Its function is non-distraction” (*Abhidharmasamuccaya*, 5th century C.E./2001, p.9). Additionally, Buddhaghosa, one of the most authoritative Theravādin Buddhist commentators of the same era (5th century C.E./1979), characterized mindfulness as “a gatekeeper because it guards the gate of the eye and so on” (p. 141). In essence, mindfulness refers to the faculty of sustained, voluntary, continuous attention focused on a familiar object without forgetfulness or distraction (Asanga, 2001; Buddhaghosa, 1979; Wallace, 2011).

At the same time, the faculty of introspection, a powerful tool for self-exploration, enables the quality control of attention, examining and explaining the components of the contemplatives' conscious experience (Wallace, 2012). Specifically, the mental faculty of introspection involves first-person exploration of the mind, to observe a broad range of mental phenomena, including our subjective experiences of thoughts, our emotions, and perceptions (Wallace, 2011). Thus, when based on rigorous contemplative training methods, inclusion of first- and second-person perspectives could yield refined phenomenological reports to improve the understanding of neuropsychological results in the study of consciousness and the mind (Lutz et al., 2008; Martiny et al., 2021; Petitmengin, 2006; Schleim, 2022).

The second-person methodological approach here refers to the reporting of observations and feedback derived from two distinct types of data collection methods. One type would include reporting from experienced meditation mentors regarding the progress of full-time contemplatives. Another type would include second-person from researcher observations and phenomenological field notes, highlighting the idea that “the researcher is the instrument” (Patton, 2002, p. 14) who provides valuable reports and data. It is crucial to emphasize that the credibility of this method depends significantly on the skill, competence, and rigor of the individual conducting the fieldwork, and it must take into account any personal distractions that may affect the researcher (Guba and Lincoln, 1981; Patton, 2002).

Thus, in the Mixed Method Research (MMR) design described here which was developed for use in a pilot study, researchers and mentors who provide the second-person data had themselves been engaging in long-term contemplative practices. That is, they had, themselves, been training their attention, mindfulness, introspection, and further, their skills in the observation and assessment of the full-time contemplatives in training. This process was based on the premise that the second-person mentors should have more knowledge and experience of meditation than the participants in the study, so they are able to detect distortions in their first-person reporting. They should also be familiar with the scientific studies that are implemented, so these mentors can also serve as intermediaries between the third-person researchers and the first-person researchers.

Apart from micro-phenomenology within the “neurophenomenological” program, there are few MMR studies on meditation, consciousness and the mind (Bitbol and Petitmengin, 2017; Berkovich-Ohana, 2017; Petitmengin et al., 2019; Varela, 1996, 1999) that explores and includes second- person reporting. One study, the Shamatha Project utilized the second-person mentor and reporting methodology (King et al., 2023; Rosenberg et al., 2015; Saron, 2013; Zanesco et al., 2021). Additionally, the participants also undertook journaling to document their first-person experiences. They submitted these daily journals to the researchers, who then conducted various third-person, psycho-physiological measurements (Rosenberg et al., 2015; Saron, 2013). This innovative methodology was integrated into the data-gathering process, but it is important to note that the second-person data collected remains unpublished.

In summary, most studies on meditation, consciousness and the mind have yet to employ mixed methods research (MMR) that rigorously combines qualitative first- and second-person data collection with quantitative measures, scales, and recordings, particularly with advanced contemplatives (Berkovich-Ohana, 2017; Huynh et al., 2019; Frank et al., 2024; Lutz et al., 2008; Northoff and Ventura, 2025; Olivares et al., 2015). This paper demonstrates how MMR was applied in *collaboration* with the researchers and the contemplatives. We incorporated first-person experiential methods for refining attention, mindfulness, and introspection (contemplation), together with second-person qualitative methods (reports) and third-person experimental data (measures). With such integration of MMR, the theoretical and methodological frameworks outlined here aimed at providing an in-depth understanding of the experience of full-time contemplatives who were highly trained in attention, mindfulness, and introspection. The main objective was to explore the nature of consciousness and gain a deeper understanding of the individual experiences of full-time contemplatives, while integrating their phenomenological experience with electrophysiological measures (EEG) and intersubjective, second-person reporting.

## Interpretative phenomneological analysis—IPA

In response to the challenges of documenting subjective phenomenological (first-person and second-person) experience, Interpretative Phenomenological Analysis (IPA) (Smith, 2024; Smith et al., 2009; Smith and Nizza, 2022) was innovatively employed as a structured methodological approach in psychology designed to reveal, analyze, and interpret contemplative practitioners' unique perspectives and meanings. IPA emphasizes the individual perspectives and meanings situated within specific contexts (Smith, 2024). This method is compatible with various qualitative and quantitative techniques, including journal writing, interviews, questionnaires, observations, and EEG and other neurological measurements.

IPA offers a depth of understanding of individual perspectives and interpretations that surpasses most other forms of thematic analysis within phenomenological psychology. It focuses on the individual's interpretation of their lived experience within a given context, involving both interviews and textual analysis (Smith, 2024). IPA also acknowledges the researcher's interpretation as part of the analysis, recognizing the inter-dynamic method between the researcher and the participant, where both the researcher and participant engage in meaning-making (Smith et al., 2009; Smith and Nizza, 2022). Indeed, IPA is particularly effective with smaller sample sizes, enabling an in-depth exploration of individual narratives. Researchers acknowledge that both the researcher and the contemplative practitioners play interpretive roles in this process. Essentially, IPA entails double hermeneutics (Smith and Osborn, 2003, p. 51), whereby participants make sense of their experiences while researchers endeavor to understand these interpretations (Smith and Nizza, 2022). As such, IPA utilizes a strong idiographic approach and transcends mere descriptions of experiences; it seeks to reveal the meanings and interpretations individuals assign to their experiences, accounting for their unique contexts and perspectives (Smith and Nizza, 2022; Smith et al., 2021). IPA allows conclusions to emerge from the data findings rather than merely testing existing theories, which is generally the focus of deductive reasoning (Smith, 2024; Smith et al., 2009; Smith and Nizza, 2022).

IPA proves especially valuable for exploring complex, ambiguous, and emotionally charged topics. Meditation and contemplative practices exemplify such phenomena: they are elusive, involving intricate psycho-somatic interactions that can be challenging to articulate. In this context, IPA is beneficial because it fosters attention, mindfulness, and introspection, enabling participants to provide a comprehensive account of their experiences. This process demands a high level of skill from the interviewer or journal reader, requiring a blend of strong empathic engagement and a finely tuned ability to delve deeper into the most pertinent aspects of the participant's narratives (Smith and Nizza, 2022). This distinctive quality makes IPA a powerful method within the landscape of qualitative research (Eatough and Smith, 2017), especially in the study of consciousness and the mind (Lawrence et al., 2024).

The theoretical and methodological framework outlined here was implemented in a small pilot study, for which the research design was ultimately guided by three questions aimed at comprehensively understanding participants' perceptions, interpretations, and awareness of their experiences. Firstly, it investigated the dynamics of attentional imbalances and how those imbalances can be overcome. Secondly, it examined the impact of long-term, full-time retreat on the quality of the contemplative subjective experiences of their mind and consciousness. Thirdly, it focused on how to identify distinct incremental stages of attentional and emotional development in individuals undertaking long term, full-time contemplative training (i.e., immersive training in attention, mindfulness, and introspection). Methodologically, these three foundational questions integrated and enriched a fourth inquiry, namely, how EEG recordings may change in response to different stages of training and types of meditation.

This paper also emphasizes that introspection, integrated within the framework of Mixed Methods Research (MMR) design, can serve as a reliable and valid instrument for the scientific exploration of the complexities inherent in the mind and the nature of consciousness. In doing so, it aspires to contribute to the body of academic knowledge by presenting a distinct application of MMR in the investigation of consciousness and the mind.

## Background of the study

All participants received attention training through mindfulness of breathing and other methods for attaining ś*amatha*—the Sanskrit term for *serenity, quiescence*, or *tranquility* (Wallace, 2006). This term also refers to a peaceful stillness of the mind in which all discursive thoughts have vanished into the space of awareness while retaining stable and vivid cognizance—as taught and practiced in the Indo-Tibetan Buddhist tradition (Wallace, 2006). Samatha training brings calm and joy to the mind and improves the “psychological immune system, making the mind less vulnerable to mental afflictions” (Wallace, 2012, p. 196). Afflictions denote a process of disturbance occurring within the mind that causes agitation, such as aversion, anger, jealousy (Tsering, 2006).

A variety of śamatha meditation techniques exist within the Indo-Tibetan Buddhist tradition. The participants in this training had the opportunity to practice mindfulness of breathing, in which they direct their attention to the tactile sensations in the body that arise in response to the breathing process. Additionally, the participants practiced a technique known as *settling the mind in its natural state*, in which their focus was on “the *space of the mind* and whatever events arise within that space” (Wallace, 2006, p. 83). These two methods were categorized as śamatha *with* a sign, where a *sign* in this context is “any object of attention that can be identified within a conceptual framework” (Wallace, 2006, p. 131). In addition, the participants also practiced a technique known as śamatha *without* a sign or *awareness of awareness*. In this method, the contemplatives simply let their awareness rest, without any referent, in its innate luminosity and cognizance (Wallace, 2006). Buddhist meditation texts commonly recommend that śamatha practice be complemented by meditative techniques known as the practice of the four *immeasurables*: loving-kindness, compassion, empathetic joy, and impartiality. These techniques contributed to the participants' cultivation of benevolent qualities that lead to ethical behavior and thus a conducive environment for training the attention. The Indo-Buddhist tradition regards these benevolent qualities and ethical behavior to be essential for training in śamatha (Wallace, 2006).

In addition to attention skills, the participants have trained in vipaśyanā or insight meditation, as found in the Indo-Tibetan Buddhist tradition (Wallace, 2011). Vipaśyanā meditation refers to contemplative science, which, for this training, guided the participants to explore the nature of their minds with insight. The contemplatives who participated in this study were also trained to investigate the nature of phenomenological reality—including the concept of “I or mine” (Wallace, 2012, p. 201), as well as feelings, thoughts, mental processes, and varieties of mental phenomena. Such training was expected to help the contemplatives cultivate compassion toward themselves and others, as well as altruism and resilience.

## The conceptual framework

In this framework, the researchers' epistemological stances influenced their research questions and selection of methodological approaches. As a result, this interdisciplinary team thoroughly examined and clarified the fundamental assumptions guiding each researcher's position at the outset of this research endeavor. The researchers built a conceptual framework by using various ways of knowing, leading to a deep understanding of the phenomena under investigation (Timans et al., 2019).

This conceptual framework derived from the philosophical doctrine of phenomenology, in which subjective experience truly matters (Frank et al., 2024). Phenomenology is a way of knowledge—a *phainomena*, or that which appears, including an internal experience of which one is aware and refers to “the data of personal experience” (Reber and Reber, 2001, p. 533). In this study phenomenology was radically participatory and involved the first-person standpoint, the openness of being in the world, and the umwelt of the contemplatives who participated in the study (Bitbol, 2019). More specifically, phenomenological psychology with its fundamental concepts—such as evidence, intentionality, intuition and intersubjectivity—was also included in this conceptual process (Spinelli, 2005; Wallace, 2001). Intersubjectivity encompasses the shared interpretations created among researchers and participants during the research process. The concept of intersubjectivity lies at the very core of Indo-Tibetan Buddhist views of the world, where an individual experience or an examined phenomenon is understood as a matrix of dependently related events, all constantly changing and dependent on each other (Wallace, 2001). In addition, this conceptual framework included hermeneutics—a philosophy and method for interpreting contemplative texts (Smith et al., 2021)—to reach a deeper understanding of the participants, who were active explorers and interpreters of their conscious experiences (Langdridge, 2018; Spinelli, 2005).

The conceptual framework also included narrative psychology (Crossley, 2000) and Buddhist psychology (Rabten, 1992; Tsering, 2006). In particular, the Buddhist Dzokchen contemplative tradition (Wallace, 2024). While narrative psychology primarily focuses on construction of meaning through storytelling and the intricate connection between language and experiences (American Psychological Association, 2025a,b; Frankl, 1992; Smith et al., 2021), Buddhist psychology provides methods for observing, developing, and transforming the mind and its enduring affective traits (Rabten, 1992; Wallace, 2006, 2005). Narrative and phenomenological psychology thus guided the research process. Buddhist psychology provided an interdisciplinary bridge or method for a deeper understanding of the contemplatives' subjective experiences and the challenges of long-term contemplative training. It provided a method to directly observe how consciousness engages with perceptual, cognitive, and affective phenomena in the space of awareness—the very space in which the engagement occurs (Kudesia and Nyima, 2015).

The quantitative information was conceptualized as “a pattern of organization of matter and energy” (Wallace, 2018, p. 33). This conceptualization served as the foundation for integration of the third—person data (i.e., the standardized questionnaires and the data acquired by electroencephalography—EEG). The overall data integration demonstrated the underlying interdependence of the first-, second-, and third-person methods. In other words, the researchers have intentionally integrated their philosophical assumptions—ontological, epistemological, axiological, rhetorical, and methodological to create the MMR (Patton, 2015; Timans et al., 2019) where phenomenology, hermeneutics and neuropsychology became equal contributors in the study design and procedure.

## Study design and procedure

Mixed methods research design is strengthened when acknowledging the distinctions between methodology, methods, and the practical steps involved in the study method and implementation. The sample design determined the sampling technique: a specific recruitment method known as emergent or purposive sampling (Patton, 2015). Specifically, the participants were selected on the basis of their background in and experience with attention, mindfulness and introspection training. The sampling frame included continuous sample adjustment to focus on what was relevant to the research questions. Participants were recruited from a group of contemplatives who were already engaged in long-term and full-time contemplative training. For example, a typical training day consisted of 6–13 h of meditation practice, depending on the stage of contemplative training the practitioners had attained. During this period, there was minimal interaction with the external world. Thus, this specific purposive sampling method was imperative for selection of the research participants who had to be able to generate and sustain attention, introspection and awareness of awareness (Frank et al., 2024). Thus, the purposive sampling technique contributed to more refined phenomenological reports and, therefore, enhanced the usefulness of neuropsychological results (Lutz et al., 2008). Moreover, this type of sampling yields rigorous qualitative data reports that impart meaning to the EEG data and expose activity patterns that would otherwise go undetected. Indeed, sample selection has been recognized as one of the most essential stages of MMR (Sharp et al., 2012). It was imperative to study consciousness with appropriately and rigorously trained participants. This type of participant selection is also known as information-rich case sampling (Patton, 2015). An essential criterion for participants was that they had had years of experience in attention, mindfulness, and introspection training and had practiced these daily. Additionally, there was a requirement for participants to be enrolled in a designated 8-week contemplative training program at the beginning of the study.

As mentioned, some of the researchers conducting the study, particularly those who contributed the second-person data, had undergone the same attention, mindfulness, and introspection training and had personal experience in the phenomena being explored. The researchers' insider and outsider positionality and reflexivity played a crucial role in the quality of sampling and importance of the relationship between the researcher and potential research participants (Braun and Clarke, 2013; Patton, 2015). However, this insider-outsider positionality in this study was flexible, dynamic, and constantly evolving, depending on the context and it impacts the potential participant's responses to the researchers. Overall, this process with clear roles, ethics and transparency was explicit in this study. It fostered trust and minimized the potential for an uneven power balance during the sampling (Patton, 2015).

After contemplatives expressed their interest in the study, the researchers contacted potential participants and booked an induction to the research to ascertain their participation in the study. This induction process created the researcher–researched relationship and established a starting point for completing the informed consent form. The process included discussions and explanations about the phenomenological aspects of the project and enabled power-sharing between participants and the researchers. Developing an equitable participant-researcher relationship allowed a genuinely respectful and collaborative research environment, credible and valid data collection (Riessman, 2008). In this context, the researchers informed and debriefed participants, listened to their questions, and later in the study, contributed to collecting ethical, comprehensive, and meaningful data (Milicevic et al., 2020). Such an approach to selecting the participants also refers to a multistage sampling design used to recruit the participants with a suitable background, making them true representatives of the population studied and who are best suited to answer the research questions (Sharp et al., 2012).

### Participants

For the first phase of this study, 20 contemplatives were invited to participate. Ten participants were assigned to the experimental group and the remaining ten to the control group. Eight participants left the study leaving twelve participants (six in each group) for the first phase of the study. As such the total number of participants was twelve (*N* = 12). Those who withdrew from the study did not want to commit to writing weekly journals for over 6 months.

The control group consisted of experienced contemplatives with a median duration of 16 years practice. They participated in an 8-week contemplative training program, but after this program they returned to their daily lives. The experimental group consisted of experienced contemplatives with a median duration of 15 years practice. They underwent the same eight-week contemplative training, but remained in full-time, long-term meditation retreat undergoing training in attention, mindfulness and introspection in solitude for years. The age range of all participants was from 25–85 years.

In the second phase of the study, six full-time contemplatives (from the experimental group) agreed to continue participating in the research protocol. In this second phase the participants agreed to accept reflective journal writing and participate in EEG recordings. In phase two, another contemplative who was in long term retreat, but did not participate in phase one, joined the study. Overall, there were seven participants in phase two (N = 7). A sample size of seven accords with Patton (2015) and Smith et al.' (2021) rationale for a relatively small number of individuals enabling a rich portrayal of phenomena studied. More specifically, when integrating the first- second - and third-person inquiry, the main concern was to fully appreciate each participant's contemplative account.

### Data collection

There were two approaches for collecting data: quantitative and qualitative data in phase one and in phase two of the study. In phase one the quantitative measure included standardized questionnaires. The rationale for utilizing these questionnaires stems from their established validation in the research literature as reliable instruments applicable to diverse participant groups. Additionally, these questionnaires have been employed in previously published studies conducted by the research team. These questionnaires were administered at the start of the 8-week training program (i.e., baseline), and, at 3 months, 6 months, and 12 months post baseline. The seven questionnaires took approximately 40 min to complete, and they were:

(1) Self-Compassion Scale Short Form- SCS-SF (Raes et al., 2011); (2) Compassion of Other' Lives Scale—COOL (Chang et al., 2014); (3) Dispositional Positive Emotion Scale—DPES—Compassion Subscale (Shiota et al., 2006); (4) Depression, Anxiety and Stress Scale−21 Items—DASS-21 (Lovibond and Lovibond, 1995); (5) The Positive and Negative Affects Schedule—PANAS (Watson and Clarke, 1999); (6) The Mindful Attention Awareness Scale—MAAS (Brown and Ryan, 2003); (7) The World Health Organization-Five WellBeing Index—WHO-5 (World Health Organization, 1998).

In phase one the qualitative data collection involved weekly, reflective journal writing from baseline. These journal entries were structured according to a set of open-ended thematic questions. These questions can be found in Appendix A. Further qualitative data included an open interview with one of the two mentors enrolled in the study. This mentor feedback provided data on participants' progress in their attention, mindfulness and introspection skills. The mentors guided the participants' contemplative progress during the study without having access to their journals or written descriptions of their practices. The mentors then provided independent observations (second-person reporting) of the participants' progress, separate from the participants' reflections (first-person reporting). The mentor interview was conducted once at 12 months post baseline of phase one. This approach fostered intersubjectivity in the study.

Four months after the completion of phase one, phase two of data collection commenced. This phase of the study was initiated as an independent exploratory phase and only included participants from the experimental group for data collection. That is, this group consisted of participants from the Phase 1 experimental group who undertook full-time, long-term, open-ended contemplative training. This research phase aimed to investigate the fourth research question. Namely, if changes in EEG recordings are associated with the different types of meditation and the development of training over time. In phase two, the quantitative data collection included EEG recordings. At baseline, the EEG recordings were taken monthly, with one recording session per participant, for the first 4 months. Following the baseline phase, the recordings were then taken in six-month intervals following the final recording at the end of the first 4 months. Six monthly recordings were taken over 2 years. Each session included a recording of a 5-min baseline resting state (R) in which a participant was sitting with eyes open, non-meditating. After this 5-min resting state condition, participants engaged in three different types of meditation, each 24 minutes in duration. The types of meditations were Awareness of Awareness (AA), Loving Kindness (LK), and Mindfulness of Breathing (MB) (Wallace, 2018, 2005, 2006). The order of the meditations was randomized within and across sessions.

Before each recording (not during) the EEG researcher conducting each session provided standardized instructions, including both written and spoken meditation guidance, to the participants. The EEG researcher remained in the room during the recordings to monitor the quality of these recordings. The researcher was strategically positioned behind the participant in a corner of the room at the desk. Each participant was positioned in the middle of the room, facing a blank wall. This aimed to minimize any visual external distractions during recording sessions. Importantly, the EEG recordings were taken within the naturalistic environment where the contemplatives resided and practiced. The EEG researcher was an experienced contemplative practitioner and a member of the contemplative community with whom the participants were familiar. The EEG researcher role was strictly limited to the acquisition of EEG recordings, and they were not involved in the subsequent analysis of the data. Such standardization of the protocol and environment, defined by consistently applied procedures, served to minimize any influences on the participants and the data collection process related to potential for the “observer effect” and the “novelty” of situation (Barto et al., 2013; Darfler et al., 2022; Huguet et al., 1999; Patton, 2015). For instance, indications of these effects may include alterations in attentional focus, heightened self-consciousness, or changes in task performance, which can potentially distort EEG data (Darfler et al., 2022). Behavioral changes were monitored through further indicators of such influences, including participants' journaling regarding their feelings about being observed, and other relevant reporting or data collected. Specifically, participants' reflective journaling occurred immediately after the EEG recording sessions.

The primary objective of this journaling exercise was to extract information pertaining to the participants' subjective experiences during the meditation sessions conducted in conjunction with the EEG recordings. The participants were instructed to carry out this journaling activity at their own discretion and in a private setting, on the same day as the EEG session. These journal entries were structured according to a set of open-ended thematic questions. These questions can be found in Appendix B.

Further data collection included observations and feedback from the EEG researcher. Notably, two distinct categories of notes were generated by the EEG researcher: Technical Notes and Researcher-Participant Notes. The Technical Notes were focused exclusively on the EEG signal quality and equipment and were not classified as data. EEG analysts utilized these notes to identify any equipment-related issues that could compromise data quality, such as noise, artifacts, and impedance mismatches. The Researcher-Participant Notes referred to the qualitative data generated by the EEG researcher in their role as a participant observer (2nd-person data), comprising phenomenological, personal observations and reflections (Patton, 2002). Following each participant EEG session, the EEG researcher responded in writing to two open-ended questions: (i) Describe your experience of being involved in the entire EEG process; (ii) Reflect on your observations of the meditators during the EEG process. Only the Researcher Participant Notes were designated as 2nd-person data and included in subsequent IPA. These second-person observational notes highlight the principle that “the researcher is the instrument” (Patton, 2002, p. 14), offering nuanced insights crucial to the research data collection, analysis and the findings. This overall data collection method enhanced the validity, reliability and credibility of the research process and provided a more in-depth understanding of the context and phenomenon being studied (Patton, 2015).

### Participant observer method

The researchers introduced a participant observer role and utilized participant observation as a method. Whilst the researchers' observations, as the second-person reports, confirmed the richness of the mixed methods, the contemplatives in this study bridged the gap between external and internal descriptions or observations and shed light on the difference between the activity of the mind, the way it functions, and its innate nature (Varela, 1996; Varela et al., 1991). Specifically, the full-time contemplatives who participated in this study had undergone years of training to understand the nature of the mind, which is different from the functions of the mind (i.e., how the brain works and how we think, feel, and behave). Namely, the contemplatives in this research had undergone rigorous and authentic training in śamatha meditation. This type of mediation is designed to develop exceptional attention, mindfulness, and introspection skills (Wallace, 2006). These skills allow the contemplatives, who were, in fact, the participant observers, to create credible, valid, and reliable direct perceptions (i.e., free of conceptual elaboration) which enable them to directly observe the nature of consciousness, the mind, and awareness (the first-person perspective). Moreover, the first-person perspective method included direct empirical observation of, and reporting on mental phenomena (i.e., the state of consciousness). These observations were reported by participants in their reflective, phenomenological journaling in phase one and phase two. These journal entries formed the transcripts that subsequently underwent IPA.

Whilst the contemplatives as participant-observers were reporting upon their own experience of consciousness, this internal process of investigation into states of consciousness, exploring their own lived experience, renders them as participant researchers (Dieumegard et al., 2021). In this study, the participants' empirical observations, awareness of awareness, introspection, and insight meditations as part of Samatha (stability of mind) and Vipaśyanā (discerning intelligence) training were used as research methodologies rather than variables measured for the impact on participants wellbeing or brain function (Wallace, 2018, 2006). This training of direct empirical observation of mental phenomena, including states of consciousness and its cultivation, was essential for studying consciousness, and the mind in this research.

Furthermore, second-person reporting was an integral part of the research process, encompassing a nuanced approach and providing a crucial basis for a thorough and insightful comprehension of the phenomenon under study. As noted, the second person observational data was collected by interviews with the participants' mentors, and observational notes by the EEG researcher. This acquisition of second-person data was based on a variety of linked theoretical models, such as the embodiment approach to psychology (Varela et al., 1991), phenomenology (Husserl, 1970), intersubjectivity (Wallace, 2001) and interdependence (Wallace, 2011). By bridging the divide between first-person and third-person perspectives in this research, second-person data collection elevated a form of triangulation, enhancing the credibility, authenticity, and conviction of the findings and conclusions (Patton, 2015). It described the lived experience through a relational process and enabled immediate and distinct intersubjective and interdependent knowing (Dieumegard et al., 2021; Wallace, 2011).

For this research, the participant observer utilized a second-person data collection method designed to go beyond evaluations, field notes, the degree of participation, or the insider and outsider perspectives (Mascaro et al., 2022). In summary, this research had three types of participant observer: (i) professional contemplatives who observed their inner processes; (ii) mentors who were interviewed on the progress of the contemplative training, and (iii) researchers who took phenomenological notes of their observations during EEG recordings.

Overall, the participant-observer's role in this research was that of a professional contemplative who has undergone rigorous training in developing and applying direct perception to examine and explore the nature of the mind (Rabten, 1992; Wallace, 2006). That is, the contemplatives viewed the nature of consciousness and the mind from within. The contemplatives who participated in this research have been trained over many years to report direct observations of the mind and consciousness that are credible, valid, reliable and verifiable (Frank et al., 2024; Rabten, 1992; Wallace, 2018, 2006). On this background, this research, therefore, considered professional contemplative practices such as meditation as a technology, which is a process or skill that involves the development and cultivation of mental faculties and the application of direct perception to observe the nature of mind (Wallace, 2018). In other words, meditation is a systematic approach to exploring and cultivating the mind and consciousness. By cultivating direct perception and insight, the participants who were full-time contemplatives–in-training provided not only the first-person data and perspective on the meditative experiences *per se*, but developed as participant researchers exploring nature of mind and consciousness. In conclusion, this study integrated first -second-third person data collection and established a unique MMR design by implementing the following:

1. Participants' introspective and reflective weekly journal writing throughout 6 months of the retreat (first-person reporting).
2. Participants' brief reflective writing exercises after each EEG recording (first-person reporting).
3. Researcher's observation (second-person reporting).
4. Mentors' observation (second-person reporting).
5. Results from standardized psychological questionnaires (third-person reporting).
6. Results from EEG recordings (third-person reporting).

**Figure 1:**
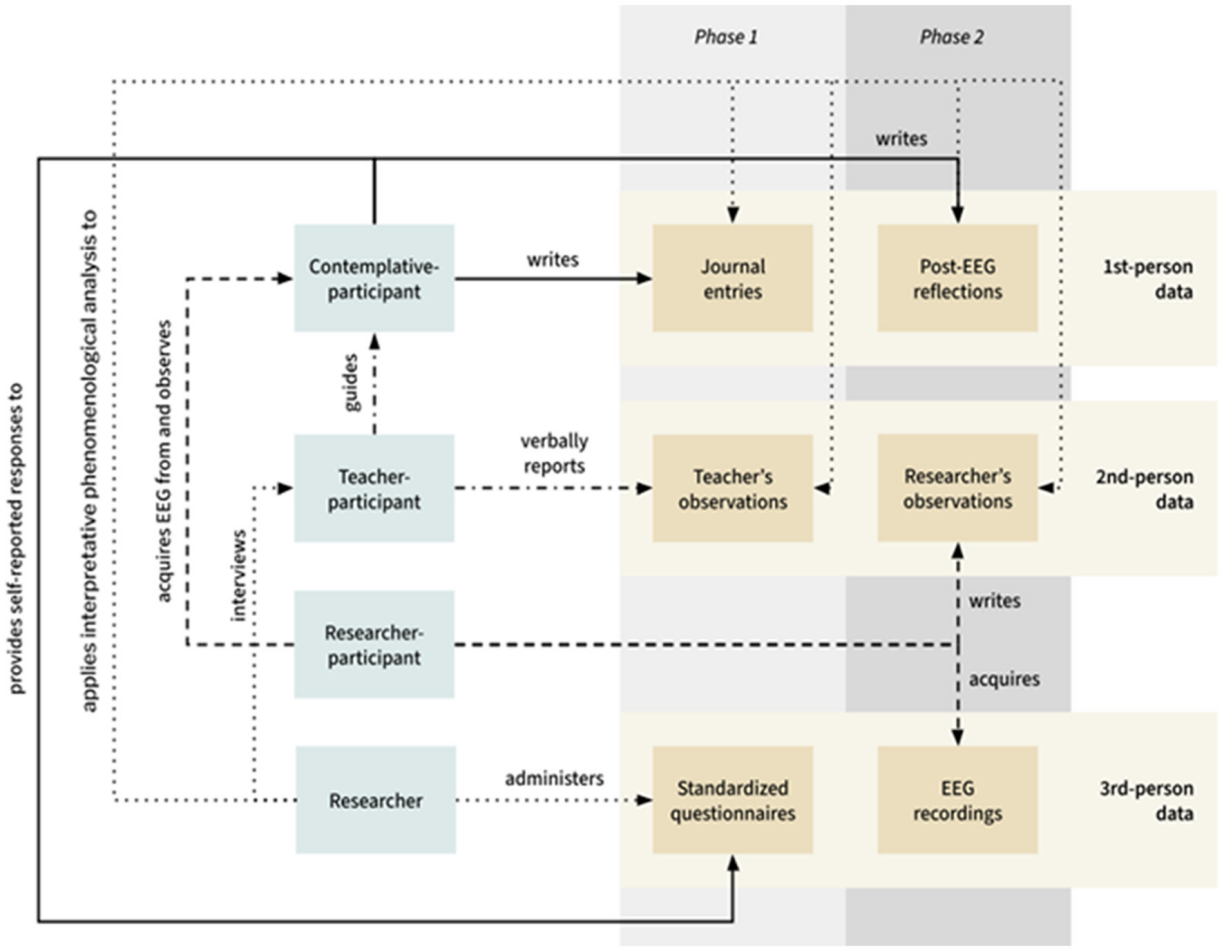
Integrated first-, second-, and third-person MMR design. The research diagram employs distinct line types to represent each research role. It only illustrates relationships within the structure of person-activity- data (read from tail to head).

Overall, intersubjectivity and interdependence as a way of knowing were central features of this applied MMR. This is illustrated in Figure 1. Integration as a meaningful two-fold process allowed the researchers to realize the benefits of mixed methods to produce meaningful, in-depth data and interpretations greater than the sum of the individual qualitative and quantitative parts (Plano Clark, 2010; Patton, 2015).

### Data analysis

Quantitative and qualitative data analyses were conducted in phases one and two of the study. The Interpretive Phenomenological Analysis - IPA focused on attentional, cognitive and emotional entities where the researchers attempted to reveal the participants' thoughts and feelings, which were then subjected to a thematic analysis (Smith et al., 2021). Hence, the IPA required an idiographic approach, where data for each participant were thoroughly analyzed in terms of their themes by initially performing a single-case analysis of the journal transcripts (Smith and Osborn, 2003). This idiographic approach began with reading, annotation, and coding of the transcript, focusing on the meaning rather than the frequency of words in the transcript. Each transcript was analyzed in detail before proceeding to the next one. The key points of the IPA analysis included a step where the researcher creates extensive notes and codes on language, tone, and mood while engaging in bracketing to acknowledge their own assumptions and expectations, and allowing for the emergence of new concepts. Subsequent re-readings were performed to identify significant issues related to key terms that influence the interpretative frameworks (Patton, 2002; Storey, 2007). The coding process was utilized to recognize connections and contradictions among key terms, relating meanings to research questions and analytical goals. Next, the researcher applied a stage of Mapping—addressing the “How” and the “What.” This stage of analysis included a holistic form-dimension (Bleakley, 2005). According to Bleakley, this refers to examining how the pattern of the story unfolds in analyzing the entire experience expressed in each journal as a narrative. The research synthesized insights and engaged in meaning-making, addressing cues of shared understanding. A key component at this stage was to define, label, and develop preliminary themes and sub-themes (Smith et al., 2021; Storey, 2007). This approach combines in-depth analytical techniques with purposeful reflection to examine participants' experiences, while also addressing the emergent hierarchy of the themes (Smith and Nizza, 2022).

In phase one, the analysis of questionnaire data examines both within and between group differences over time. Directional hypotheses were set within each of the two groups (i.e. experimental and control groups) and between these groups across the data collection points. *T*-tests are used to examine expected differences between and within groups. Any concerns about the small number of participants were mitigated by having directional hypotheses and having two stable participant groups.

EEG recordings were acquired using a 24-channel research EEG system (Mobi24) with electrodes mounted in standard 10–20 locations, and electrode impedances of 10K ohms or less. The data was imported to EEGLAB 2019.1 and pre-processed for removal of bad channels and band pass filtered at 1–55 Hz. This was followed by manual artifact rejection, spherical interpolation for replacing rejected channels, ICA decomposition and removal of IC's corresponding to eye movement or muscle activity. A final review for manual removal of any remaining artifact. The cleaned data files were included in a Study structure within the EEG lab with information regarding condition and session for each epoch. Global processing was used to calculate Σ, Φ and Ω for each epoch, and the mean values for the global variables were then calculated for each session.

Global EEG analysis was applied as the initial method for quantifying brain functional states using three descriptors derived from multi-channel sampling of the electrical field of the brain appearing on the scalp (Wackermann, 1999). This method was considered to be a holistic description of the brain's activity, allowing access to patterns of whole-brain electrical activity. The descriptors were global field strength Σ, generalized frequency Φ, and spatial complexity Ω. These descriptors form a three-dimensional system for mapping the trajectory of brain “macro-states” and have been applied in sleep research, clinical neurophysiology, psychopharmacology, perceptual and cognitive processes, and more (Gao et al., 2018; Wackermann, 1999; Wackermann and Allefeld, 2007). Typically, global descriptors are calculated across segments of EEG ranging from two to eight seconds in length, thus compressing the multi-channel EEG data to only three quantities per segment. For the purposes of this research, EEG recordings for the participant were divided into 4 s epochs and the global descriptors for each segment were calculated. Then for each session the means of each global descriptor were obtained.

The later phase of the EEG analysis also included EEG microstates segmentation, in which the EEG signal was represented as a series of stable and recurring topographic patterns to study the participants' internal states on a millisecond scale (Koenig et al., 2002) which resulted from the synchronized activity of spatially distributed electrical sources (Delorme and Makeig, 2004; Khanna et al., 2015).

In summary, the IPA findings, systematically conducted across both phases 1 and 2 at set intervals, provide a comprehensive subjective account of changes over time (Smith and Nizza, 2022). The IPA analyses would be combined with the quantitative data from phase 1. Furthermore, the IPA findings in phase 2 would be compared with the quantitative data (EEG). The integration of the qualitative and quantitative findings from phase 1 would then be compared and contrasted with systematic qualitative and quantitative data from Phase 2, where applicable and by the types of data.

## Integrating the analysis

The process of integration, a cornerstone of MMR, was of paramount importance in this research. It involved synthesizing insights from diverse sources to gain a comprehensive understanding of the phenomena under investigation (Uprichard and Dawney, 2019). This approach transcended the limitations of a single method or paradigm, adopting a holistic perspective that incorporated the integration of data and outcomes (Bazeley, 2011; Patton, 2015). In this research, integration has been implemented at various levels, including epistemological, design, methods, and analysis, across all three types of data (the first, second, and third person). This comprehensive integration strategy has been instrumental in complementing and confirming the results and findings of each component of the research process. More specifically, such integration included the qualitative approach—the Interpretative Phenomenological Analysis (IPA), concerned with the detailed examination of personal lived experience, the meaning of experience to participants and how they make sense of that experience (Busetto et al., 2020; Dieumegard et al., 2021; Milicevic et al., 2020; Smith et al., 2021). IPA also included the analysis of transcripts from one-to-one interviews with the mentors by the researcher and the EEG researcher observation notes (the second-person data). The interviews revealed the mentors' observation of and feedback on participants‘ progress.

Thus, the IPA was committed to exploring the linguistic constructs and meanings throughout the narrative and journal writings. The aims and research questions of this study were designed to focus on specific contextual expertise while remaining intentionally open and exploratory (Smith et al., 2009; Smith and Nizza, 2022). The following example presents a case study and provides high-level insights into a segment of data integration from Phase 2 of the pilot study. The integration process comprised two stages. First, the results obtained from IPA coding and thematic analysis over the four months period. The second stage demonstrates how the IPA themes, subthemes, and codes were corroborated by EEG data and the types of meditation practiced over time.

### IPA findings—Stage 1

The findings reveal aspects and dimensions of the participants' experiences, as journaled in solitude, immediately after the EEG recording sessions. A central experiential theme that emerged from these qualitative findings was Interdependence. The theme of Interdependence emerged as a superordinate theme, providing a framework for the context of other experiential themes and subthemes. Interdependence consisted of three main themes: (1) Conation, (2) The Meaning of Life, and (3) Resurgent Attention, along with a subtheme, (4) Eudaimonia. Collectively, these themes reflect the participant's desires, discernment, introspection, attentional qualities, cognitive, emotional, and spiritual balance. The findings demonstrated potential benefits and underscored the Interdependence between contemplative practice and the experience of genuine well-being, as well as exploring the nature of the mind and its relationship to reality.

Further phenomenological information was collected as part of the ongoing process during the EEG recordings, which included observations from the resting state. The researchers adapted and enriched their method by adding 'resting state' related questions into the participants' journal. The experiential themes about the resting state were vital for a refined understanding of the EEG results and their complexity. After the above IPA findings (Stage 1), the text structure was further organized by meditation type within each recording session. The intention was to gain a more in-depth understanding of the phenomenological data so that it can be readily integrated with the structure of the EEG analysis. The overall findings from this further coding process (Stage 2) were consistent with the Stage 1 original IPA findings presented in Table 1.

**Table 1:** The themes and sub themes emerged through analysis.

**Thematic level**	**Code**	**Themes**
Superordinate theme	1.0	Interdependence
Master theme	1.1	Conation
Master theme	1.2	The meaning of life
Master theme	1.3	Resurgent Attention
Subcategory	1.3.1	Eudaimonia

Code indicates thematic hierarchy of themes and corresponds to the four relevant EEG recordings. For further thematic illustration, please refer to Appendix C, which contains specific examples and quotations that exemplify the emerging themes.

### Integrating IPA findings with EEG results—Stage 2

The integration and further IPA coding as corroborated by the EEG data, are illustrated in Table 2. This table presents the coding process in accordance with EEG recordings by mediation type over time.

**Table 2:** IPA coding by EEG mediation type.

**Session**		**Coding**		**Emergent themes**
	**amp freq cmplx**			
September	AA 7.2 13.3 2.6	MB 7.4 12.9 2.9	LK 8.5 16.2 3.1	
	Inspiration/joy	First EEG/intention/discernment	Conation	Intentionality
October	AA 6.8 16.0 5.0	MB 8.4 13.6 3.2	LK 8.7 13.4 3.2	
	Introspection	Continuity/meaning/focus	Conceptual mediation	Meaning
November	MB 8.0 13.8 3.3	AA 8.0 13.3 3.0	LK 7.8 12.0 2.7	
	Continuity/bliss	Resurgent attention	Mindful loving kindness	Purpose/eudaimonia
December	MB 7.1 13.4 2.9	LK 7.8 14.1 2.6	AA 8.8 13.0 2.6	
	Tranquility/peace	Eudaimonic conation	Continuity/less cognition	Interdependence

In the second phase of the study, the IPA was a revealing analytical technique that informed and guided the EEG data analysis and emphasized the active role of the researchers. Specifically, the EEG data analysis had a hierarchical structure. That is, the repeated measures were always nested for the participants. The outcomes of the EEG analysis were integrated with findings from the first-person reflective journal writing (by the participants) as well as second-person IPA outcomes (based on the EEG researcher' observational notes). This integration offered a new perspective and interpretation of the comprehensive findings. Integrating the second-person IPA findings contributed to a more profound comprehension of the contemplatives' progress in their attention, mindfulness, and introspection. It facilitated intersubjective validation between the mentor and contemplatives, emulating the validation method employed within the meditative tradition of Indo- Tibetan Buddhism for millennia (Frank et al., 2024; Rabten, 1992; Varela et al., 1991; Wallace, 2006).

### Expansion and the anticipated results

On multiple occasions, the research revealed the importance and complexity of integrating the first-, second-, and third-person MMR approaches. For example, in one instance, analysis based on the mean global values for each meditation session—assessing relative amplitude, frequency and complexity (Wackermann, 1999; Wackermann and Allefeld, 2007), suggested that the participant's EEG readings were indicative of heightened cognitive activity. Specifically, during this meditation session, the EEG displayed frequencies in the beta range accompanied by a complex topographical pattern. It contrasted with other sessions in which the same participant exhibited the lowest frequency and second-lowest complexity values across all recordings, consistent with alpha-range activity, and interpreted as reflecting a more relaxed neural state (Deolindo et al., 2020). The presence of beta-range frequencies and high complexity raised questions about whether the participant was genuinely in a meditative state, as such patterns typically diverge from the neural signatures associated with calm, focused awareness.

During the same session in which EEG readings suggested heightened cognitive activity, the EEG researcher reported the participant's slow physical movement, which appeared to be, in their assessment, due to discomfort from the experimental cap. In contrast, the first-person phenomenological data revealed that the participant was experiencing a deep meditative state. The participant self-reported being aware of and had intentionally incorporated the EEG experiences, including discomfort, into the meditative practice. Notably, the first-person data analysis revealed that the participant had cultivated a mindset of impartiality toward all phenomena being experienced and observed during this meditation. Despite the complexity of this meditation session, the EEG recording did not capture a meditative experience. Such instances have resulted in so-called expansions when the employment of multiple disparate methods allows one to expand insights, even when those methods yield divergent findings (Fetters et al., 2013).

Importantly, such expansions in understanding can occur not only with respect to the analysis or interpretation of one's data but also with respect to the application of one's methods. In other instances, the integration of analyses guided and helped to confirm the original findings of a subset of data (Fetters et al., 2013). For example, as illustrated in Table 2, the emergent themes of the IPA analysis were compared with the EEG analysis. Furthermore, the EEG analysis guided a different approach to the IPA analysis. The coding and emergent themes were explored by meditation type across the four recording sessions. The emergent themes of these two approaches to the IPA analysis resulted in the same superordinate theme.

In another example, the participant was found to be in a state of deep meditation while simultaneously experiencing a high volume of conceptual phenomena. This observation prompted a closer look at the EEG results, with the potential to examine the relative activity of brain regions in reference to large-scale neural networks (Menon, 2023). The aim was to elucidate the cognitive neuropsychological findings.

An additional example referred to the integration of the results demonstrating no difference between the EEG resting states and meditative states in these advanced contemplatives. As a result, the experimental design was revised to include phenomenological reporting and analysis of the resting state. This integrative MMR process is recognized as expanding insights or confirming findings (Fetters et al., 2013). All approaches to the analysis were brought together with the assumption that integration is both theoretically and practically feasible, as long as epistemological differences can be bridged and integrated into underlying interdependence and coherence.

## Discussion

This paper contributes to the literature on the study of consciousness by demonstrating integrative MMR to study the mind, particularly attention, mindfulness and introspection (Gotink et al., 2015; Huynh et al., 2019; Mascaro et al., 2022; Rich et al., 2022). Importantly, this MMR design and procedures have illustrated the necessity of including first - person reliable, credible and valid direct observation of consciousness itself and the mind (Bitbol and Petitmengin, 2017; Berkovich-Ohana, 2017; Frank et al., 2024; James, 1950/1890; Medeiros et al., 2021; Petitmengin, 2006; Schleim, 2022; Varela, 1996; Wallace, 2018, 2006).

### Contribution to mixed methods research and consciousness research

This article contributes to the MMR design which is embedded in the interdependence of knowledge (Mascaro et al., 2022; Spinelli, 2005; Wallace, 2001), revealing how each conceptual framework in the respective discipline could be flexible, inter-dynamic and ultimately integrated. It is imperative to emphasize that the aim to investigate consciousness and the mind has determined the methodology, methods, and the participants. We have put forward an overview of the significance of integrating first-, second-, and third-person perspectives (Achepohl et al., 2022; Busetto et al., 2020; Huynh et al., 2019; Lumma and Weger, 2023; Mascaro et al., 2022). This approach aligns with a methodological study of consciousness which necessitates participants who have extensive training to generate and sustain attention, mindfulness and introspection in the study of consciousness and the mind (Frank et al., 2024; Lutz et al., 2008; Wallace, 2012).

This integration, as a meaningful two-fold process, allowed the research to harness the benefits of MMR, producing profound, in-depth data and interpretations that surpass the sum of the individual qualitative and quantitative parts (Katyal and Goldin, 2021; Patton, 2015; Plano Clark, 2010; Posner et al., 2014; Yoshida et al., 2020).

The IPA findings have enriched the interpretation of the EEG results bringing meaning and context to the participants' contemplative practice and insights into their experience of consciousness and the mind. The interpretation of the synthesis of the EEG and IPA results have elicited further qualitative analysis. Despite the potential benefits of such an integration, more research is needed in this area. Further investigation into this type of integration could lead to new insights into the relationship between the researcher and the research participants, enabling further development of MMR (Hut and Shepard, 1996; Kudesia and Nyima, 2015; Varela, 1996). To this end, it is essential that researchers are aware, transparent, and flexible about their philosophical assumptions (Braun and Clarke, 2013; Reber and Reber, 2001; Riessman, 2008). This includes their assumptions about ontology, epistemology, axiology, rhetoric, and methodology (Patton, 2015; Timans et al., 2019). By doing so, each perspective and scientific view can contribute equally to the study design and procedure, resulting in a well-rounded and comprehensive scientific outcome.

Furthermore, this paper illustrates the importance of a specific purposive sampling method as instrumental in meeting the research's need for participants who could contribute to more refined phenomenological reports and enhance the usefulness of neuropsychological results (Patton, 2015; Sharp et al., 2012; Lutz et al., 2008; Varela, 1996). The purposive sampling method was used in this research to select participants with the ability to generate and sustain attention, mindfulness, and introspection. The researchers employed information-rich case sampling within their insider and outsider positionality and reflexivity (Braun and Clarke, 2013). This process was imperative for maintaining trust and equality of the power balance crucial for the in-depth study of consciousness. In this paper, we have outlined the faculty of introspection as a reliable, verifiable, and credible tool for self-exploration, and as the quality control of attention which in turn examines the components of the long-term, full-time contemplative experience (Wallace, 2012). This paper argues that introspection can be considered a trainable subjective skill, as has been understood in the Indo- Tibetan Buddhist contemplative tradition (Frank et al., 2024; Lutz et al., 2008; Wallace, 2012). A skill that can imminently be incorporated into MMR design. This design lends itself to the formulation of research protocols investigating the nature of consciousness and the mind itself. It is further posited that consciousness and the mind, as they are understood in the Indo-Tibetan Buddhist tradition, can in the context of scientific inquiry, be considered as phenomena in and of themselves, not necessarily as inherent epiphenomena of brain function (Josipovic, 2021, 2024; Laukkonen and Slagter, 2021; Metzinger, 2020, 2024; Sattin et al., 2021). Specifically, they have identifiable characteristics which can be directly observed and reported on by those who have trained and sustained skills in introspection, attention and mindfulness and therefore can be investigated through scientific inquiry.

More research is required on the direct empirical observation of consciousness and its inclusion in MMR. The inclusion of direct empirical observation by way of first and second-person reporting enables researchers to open themselves to the research experience, developing new concepts with an exploratory approach rather than expectation to prove any theoretical framework (Busetto et al., 2020; Fetters et al., 2013; Milicevic et al., 2020; Smith et al., 2021). In this way, the third—person reporting of the results from EEG recordings (Deolindo et al., 2020; Khanna et al., 2015; King et al., 2023; Koenig et al., 2002; Menon, 2023; Rosenberg et al., 2015; Saron, 2013; Wackermann, 1999; Zanesco et al., 2021) goes beyond highly specialized but isolated interpretations and becomes a segment of intersubjectivity and interdependence as a way of knowing and understanding the studied phenomena (Husserl, 1970; Uprichard and Dawney, 2019; Wallace, 2001).

## Limitations and conclusions

Bringing multiple, seemingly disparate methods together can teach us not only about data but also about these methods, including their limitations. For instance, the purposive, information-rich sample was recruited from a population within one contemplative tradition; as such, the findings cannot necessarily be generalized to contemplatives across different traditions worldwide. However, limiting the participation to highly trained individuals within a single tradition allowed for precise reporting and in-depth analysis of the phenomena being investigated. Another limitation was the large age range and different cultural and professional background of the contemplative participants. However, the sample nonetheless achieved cohesion by the fact that all the participants were advanced contemplative practitioners on entering the study. A further possible limitation was that phase one of the study included only one interview with the mentors because of their limited availability while in their own meditation retreats. However, this interview was purposely scheduled at the end of phase one of the study so that the mentors would have greater insight regarding the progress of their students. In addition to the above considerations, it should be noted that many of the researchers were also contemplative partitioners. As such this contributed to the MMR design and the participant—researcher role (Dieumegard et al., 2021; Mascaro et al., 2022).

In summary, this paper aimed to demonstrate the necessity of integrated MMR design in the study of consciousness and the mind. Within this design we emphasized the importance of reliable and valid first-person perspectives in integrating second- and third -person research methods to serve as a touchstone for advancing understanding of consciousness and the mind. The meaningful understanding of research on consciousness can only come from interdependent knowledge across seemingly diverse disciplines. Historically, this is not an unusual approach (Tsering, 2006). Though it has been overlooked (Bitbol, 2019; Bitbol and Petitmengin, 2017; Frank et al., 2024; Josipovic and Baars, 2015; Lutz et al., 2008; Varela, 1996; Varela et al., 1991; Wallace, 2018, 2012). By outlining this approach, we invite fellow researchers to further our knowledge through collaboration.

## Data Availability

The datasets presented in this article are not available due to ethical, legal, and privacy considerations. The data supporting the pilot study's findings are unavailable. This limitation is in accordance with the ethical consent agreement signed by the participants. For any additional inquiries, please direct your communications to the corresponding author, Anita Milicevic. anita.milicevic@centerforcontemplativereserach.org.
