# Supplementary material for: Consciousness, mindfulness, and introspection: integrating first- and second-person phenomenological inquiry with experimental and EEG data to study the mind
Source: Front Psychol. 2025 Sep 8;16:1558453. doi: 10.3389/fpsyg.2025.1558453 (PMC12450712; doi:10.3389/fpsyg.2025.1558453)
Supplement: Supplementary file 1 [file Supplementary_file_1.pdf]

Appendix A: PHASE 1- OPEN-ENDED THEMATIC QUESTIONS FOR JOURNAL WRITING

Weekly reflections on the practice:

1. What feelings were generated by your practice?

2. What aspirations were generated by your practice?

3. What thoughts/insight stood out for you?

4. What bodily changes or states were you aware of at the time?

5. How your cultivation of attention has evolved this week?

6. How did your experiences affect you? How did you respond?

7. Please tell us the methods you have used to train attention.

8. What changes do you associate with the experience?

9. What do you most value about your practice now?

Monthly reflections on the practice:

1. Please describe in what ways, if any, you have applied compassion and loving-kindness practice during the last 4 weeks?

2. What role does empathetic joy play in your practice?

3. Please describe in what ways, if any, have you applied equanimity practice during the last 4 weeks?
