# Supplementary material for: Consciousness, mindfulness, and introspection: integrating first- and second-person phenomenological inquiry with experimental and EEG data to study the mind
Source: Front Psychol. 2025 Sep 8;16:1558453. doi: 10.3389/fpsyg.2025.1558453 (PMC12450712; doi:10.3389/fpsyg.2025.1558453)
Supplement: Supplementary file 2 [file Supplementary_file_2.pdf]

APPENDIX B: PHASE 2 - OPEN-ENDED THEMATIC QUESTIONS POST EEG

Meditation 1:

Would you please describe your experience of meditation during the EEG?

What feelings were generated?

What bodily changes or states were you aware of at the time?

What thoughts, if any, occurred?

Meditation2:

In what way does your experience during this EEG differ from previous experience?

In what ways, if any, this experience has affected you?

What is your reason for participating in this study?

Meditation 3:

Please describe your experience of meditation during the EEG.

In what way does your experience during this EEG differ from previous experience?

What feelings were generated?

What bodily changes or states were you aware of at the time?

What thoughts, if any, occurred?

Resting state:

Please describe your experience of resting state.

In what way does your experience during the resting state differ from the meditation experience?

What feelings were generated?

What bodily changes or states were you aware of at the time?

What thoughts, if any, occurred?
