# Supplementary material for: Consciousness, mindfulness, and introspection: integrating first- and second-person phenomenological inquiry with experimental and EEG data to study the mind
Source: Front Psychol. 2025 Sep 8;16:1558453. doi: 10.3389/fpsyg.2025.1558453 (PMC12450712; doi:10.3389/fpsyg.2025.1558453)
Supplement: Supplementary file 3 [file Supplementary_file_3.pdf]

Appendix C: Thematic Analysis and Sub-themes from IPA, with Participant Excerpts

1. **Conation**

The experience of the first EEG recording was an overall sense of heightened awareness with effortless, open monitoring of the participants' thoughts, feelings, and overall mental experiences. This experience has been termed – meta-awareness or monitoring awareness - the capacity to observe one's thoughts and feelings without identification, distortion, and with kindness (Dimidjian & Thupten, 2018). In the first recording the participant described this meditative state in the following way:

My experience was actually quite pleasant, and I did not feel bothered by the equipment. I love the meditation I practiced in this session, and I practice it quite often on my own, so I found it basically just another pleasant meditation session. That said, I did feel a slight overall heightening of awareness and intensity—a heightening in luminosity of awareness—during the session…. Lots of luminosity and intensity of awareness as well as an ongoing sense of peace, stillness, sometimes bliss and wellbeing. Inspiration and joy arose at times as well.

During the first session, the participant's meditative state was described as a deep sense of relaxation and high focused and firm intention. It appears that emotions of joy erose when experiencing the novelty of a situation. That is heightening intention and focus on the context of participating in the contemplative research protocol and the first experience of the EEG recording. The term novelty refers to mental phenomena involved in a cognitive process that elicits attention and strong emotions (Barto et al.,2013; Lisman & Grace, 2005). For example, the participant wrote:

It was just a nice meditation experience and somewhat thrilling and delightful to be doing my first EEG reading.

Exposure to novel experiences is compelling when coinciding with positive goal congruence. The participant exposure during the first EEG recording was in accord with the participant's intentionality as an essential feature of mental or cognitive states (Reber, 2011). For example, the participant's desire to participate in the study was expressed in the following way:

I believe that ongoing collaboration between contemplatives and scientists can lead to exciting breakthroughs in our understanding of mind and consciousness and I think such breakthroughs can lead to great benefits for society.

All the above refers to connection as our faculty of desire and conative balance as crucial elements of mental health. The participant's conation was described in the following way:

Mostly feeling of bliss, joy, and happiness—with also some sense of soaring inspiration. I noticed blissful and pleasant bodily sensations all over, but particularly in the heart areas and up through the spine to the crown of the head. I also experienced some warmth in the heart areas and a bit throughout the body.

When describing feelings that were generated during the first EEG recording, the participants wrote:

I was consciously generating through loving kindness and well-wishing for myself and others and others… I did not find myself distracted or bothered by the equipment and as this meditation is particularly beautiful and heart-warming (loving-kindness) I had a very lovely and even blissful experience.

2. The meaning of life

The meaning of life is inseparable from the ethical way of life (Frankl, 1992), committed to cultivating mental health and balance while fathoming the depths of our innermost nature as the source of genuine well-being (Wallace, 2015). Continuity and commitment to the practice as the meaning of life were evident in the second EEG recording session and described in the following way:

To me it just felt mostly like another meditation session. And as I meditate basically all day every day and I consider it the most meaningful thing to do at this moment in my life, I simply enjoyed these sessions as another opportunity to practice what I love and further cultivate my mind in beneficial ways. ... Overall, I am committed to the collaboration between contemplatives and scientists as a means to bring to light new understanding of the mind, consciousness, and their role in reality as a whole.

In the second session a month later, the participant is articulating the continuity of their practice over time, and it is becoming meaningful and essential. While reflecting upon the commitment and meaning of life, the participant reported two or more parallel psychological and emotional processes. For example, the participant became more acutely aware of being observed – quote:

It was very pleasant. All of the practices are very familiar to me and dear to my heart, so it is a joy to practice them, including the one chosen for this session. Of course, there is some sense of being "watched" and there is a mild discomfort wearing an EEG cap, but

neither of these affected me very much. Overall, the sense of being in a "reading" only really heightened the vividness and acuity of my awareness I believe.

The extract presented above shows the complexity of the participant experience, including awareness of being observed dividing the participant's attention between nonconceptual resources and conceptual – thinking. This divided attention can be defined as the type of simultaneous attentional processes that allows one to manage two or more channels of information simultaneously so that tasks and experiences can be attended to concurrently (APA, 2023). For instance, the participant reported:

In this meditation, thoughts are used like paper, kindling, and firewood to start the fire of the loving-kindness meditation. Once the thoughts do their job of generating the feeling and inner sense of the meditation, then mostly it is just visualization of light and so on. So mostly, aside from the occasional random thought, all the thoughts in this meditation are connected to either directing wishes of loving kindness to myself or to others.

And then further experience of meditation during this EEG recording:

Once again, it was a pleasant and familiar meditation experience, with the exception of wearing the cap and having a clear sense of being monitored. That said, within the context of the meditation, very little of my awareness is focused on the setting of the meditation and I am mainly focused on the content of the meditation, which is very familiar to me. This session was on loving-kindness, which is a beautiful meditation to do and so I thoroughly enjoyed the session.

3. Resurgent Attention

Resurgent attention refers to the stage of attentional stability where the partitioner remains engaged in the continuity of practice; the mind remains focused on the object of mediation most of the time in all sessions (Wallace, 2006). The excerpts from the participant's journal in the third EEG recording session suggest stability and continuity of practice. For instance:

The practice was mindfulness of breathing. I love this practice, especially when practiced in this style with an emphasis on an open and free awareness that does not collapse down to the sensations or reify a sense of a "body." The practice was very soothing, peaceful, and brightly luminous and in that sense, somewhat thrilling. It also had a spacious quality to it which leads to a greater sense of ease.

I felt very calm, peaceful, and at ease. At the same time, there was also a sense of joy and a light blissful quality. In addition, there is the ongoing brilliance and vividness of awareness knowing itself, which adds the sense of a thrilling experience.

Overall, I did not have much awareness of my body other than an overall openness and soothing quality. I did not experience much of any "rough borders" or pains in the body. It was more of just a soft looseness and a melting quality. There is also an experience of energy in the spine that has a way of "lifting" the energy and body upwards, and it seems to support the whole spinal column, such that not much muscular activation is required.

As thoughts were not my focus in this meditation, I don't recall specific thoughts that arose. Though I do recall that from time-to-time thoughts about the practice itself would arise, either related to quality control (introspection) or remembering (mindfulness) the instructions for the practice. There also arose from time-to-time thoughts about random things, but these were mostly just bubbles that arose and dissipated without much content.

In this stage of contemplative practice, the more relaxed, stable, and clear the attention, the deeper the mind and the body relax. This state of meditation is thought to aligned with an egoless or selfless quality of the participant's sense of well-being (Wallace, 2006). For example,

I'm just resting there being, and in that same stream of being, effortless being aware of being aware. In this way, there is almost a sense of the practice "doing itself," while [the] "I" just get to rest in inactivity. In this way, there is a great sense of openness, expansiveness, and brilliance, which leads to experiences of joy and happiness.

Overall, there was not much experience of my body, other than just a soft grounding presence, but with very little sense of borders or hardness. In this way, the bodily experience is soothing and blissful, especially as the relaxation gets deeper and deeper. There is also an experience of energy in the spine that has a way of "lifting" the energy and body upwards, and it seems to support the whole spinal column, such that not much muscular activation is required.

4. Eudaimonia

The key to human flourishing is the cultivation of genuine well-being, what the ancient Greeks called "eudaimonia." It requires cultivating exceptional mental health and balance and gaining insight into the meaning of life and the nature of existence (Wallace, 2005). The data gathered in session four suggest that the participant experienced a sense of

genuine well-being and importantly a deep desire and intention to continue to cultivate the same. For example:

If anything, I did notice a bit of a deeper and more expansive love and joy coming into my being, which had a noticeable subtle physiological effect. I felt more energy rushing into the spine and up through the heart and into the crown of the head at times.

As usual, this practice of loving-kindness inspires me and brings me great inspiration and joy. It makes me want to practice loving-kindness even more and to suffuse my whole being with this heart quality of vast and deep loving-kindness.

In summary, over the four sections, Interdependence of the participant's Conation, The Meaning of Life, Resurgent Attention and Eudaimonia, and the Collaborative Method of this inquiry, was demonstrated in the following journal entry:

It is my hope that through this collaboration between scientists and experienced contemplatives we may bring to light in a scientific way new insights and understandings of mind and consciousness as well as new methods for studying the mind and consciousness.

These findings align with the profound reality of connectedness that is articulated by the Buddhist principles of the interdependent nature of reality (Dalai Lama, 1993). This forms the foundation of contemplative science.
